# ACE2 deficiency increases NADPH‐mediated oxidative stress in the kidney

**DOI:** 10.1002/phy2.264

**Published:** 2014-03-24

**Authors:** Jan Wysocki, David I. Ortiz‐Melo, Natalie K. Mattocks, Katherine Xu, Jessica Prescott, Karla Evora, Minghao Ye, Matthew A. Sparks, Syed K. Haque, Daniel Batlle, Susan B. Gurley

**Affiliations:** ^1^ Department of Medicine Division of Nephrology and Hypertension The Feinberg School of Medicine Northwestern University Chicago Illinois; ^2^ Department of Medicine Division of Nephrology Duke University and Durham VA Medical Centers Durham North Carolina

**Keywords:** ACE2, kidney disease, reactive oxygen species, Renin–angiotensin system

## Abstract

Angiotensin‐converting enzyme 2 (ACE2) is highly expressed in the kidney and hydrolyzes angiotensin II (Ang II) to Ang(1–7). Since Ang II is a strong activator of oxidative stress, we reasoned that ACE2 could be involved in the regulation of renal oxidative stress by governing the levels of Ang II. We, therefore, assessed levels of oxidative stress in kidney cortex of ACE2 knockout and wild‐type littermate mice under baseline conditions. We found multiple markers of increased oxidative stress in ACE2KO mice. NADPH oxidase activity was increased in kidney cortex from ACE2KO mice as compared to WT (227 ± 24% vs.100 ± 19%, *P* < 0.001). However, kidney catalase and superoxide dismutase activities were not different between groups. Exogenous Ang II was degraded less efficiently by kidneys from ACE2KO mice than WT mice, and administration of an AT1R blocker (losartan 30 mg/kg/day) resulted in normalization of NADPH oxidase activity in the ACE2KO. These findings suggest that an AT1R‐dependent mechanism contributes to increased ROS observed in the ACE2KO. This study demonstrates that genetic deficiency of ACE2 activity in mice fosters oxidative stress in the kidney in the absence of overt hypertension and is associated with reduced kidney capacity to hydrolyze Ang II. ACE2KO mice serve as a novel in vivo model to examine the role of overactivity of NADPH oxidase in kidney function.

## Introduction

The renin–angiotensin system (RAS) is a key regulator of blood pressure and cardiovascular function. Upregulation of the RAS, mainly through the interactions of Angiotensin II (Ang II) with the Angiotensin II type 1 receptor (AT1R), can lead to hypertension and end organ damage (Ito et al. 1995). Angiotensin‐converting enzyme (ACE) inhibitors and angiotensin receptor blockers (ARBs), which prevent the production or action of Ang II, respectively, are potent antihypertensive agents associated with reduction in cardiovascular disease burden, independent of their BP lowering effect (Lewis et al. 1993; Yusuf et al. 2000; Brenner et al. 2001; Dahlof et al. 2002).

Angiotensin‐converting enzyme 2 (ACE2) is a monocarboxypeptidase that degrades Ang II and can, therefore, regulate the RAS (Donoghue et al. 2000). ACE2 is highly expressed in the kidney, which suggests that it may play a role in regulating renal function both in health and disease (Donoghue et al. 2000; Vickers et al. 2002). ACE2 knockout (ACE2KO) mice have markedly enhanced susceptibility to Ang II‐induced hypertension but baseline blood pressure levels are normal or only slightly elevated (Gurley et al. 2006). ACE2 has previously been shown to play a role in kidney disease (Ye et al. 2006; Soler et al. 2007, 2013) hypertension (Yagil and Yagil 2003; Gurley et al. 2006), heart failure, (Crackower et al. 2002; Huentelman et al. 2005), inflammation, (Thomas et al. 2010) and atherosclerosis (Dong et al. 2008).

In the initial description of ACE2 knockout mice, the dominant phenotype at baseline was a marked defect in cardiac contractility, which was more severe in older male ACE2KO mice and also accompanied by severely reduced blood pressures (Crackower et al. 2002). Yamamoto et al. (2006) later generated a separate line of ACE2‐deficient mice, and in contrast to the findings by Crackower et al. (2002), their line of ACE2KO mice had normal baseline cardiac function and morphology, but showed increased susceptibility to a pressure overload model of heart failure. In the line of ACE2‐deficient mice generated by our group (Gurley et al. 2006), on both mixed and inbred genetic backgrounds, we observed normal cardiac dimensions and function. The ACE2KO mice on a C57BL/6 background had slightly higher systolic BPs at baseline compared to their WT littermates (~7 mmHg) (Gurley et al. 2006). This difference in blood pressure, however, was not observed in 129/SvEv mice, suggesting that the contribution of ACE2 deficiency to baseline BP regulation may be determined in part by the genetic background and that compensatory mechanisms are set in play that prevent overt hypertension (Gurley et al. 2006).

Deficiency or downregulation of ACE2 leads to an impaired ability to degrade Ang II, leading to the accumulation of this biologically active octapeptide particularly in pathological conditions where the RAS is upregulated (Gurley et al. 2006; Wysocki et al. 2010; Ye et al. 2012). Increased levels of Ang II promotes local tissue damage by several actions including vasoconstriction, inflammation, fibrosis, oxidative stress, DNA damage, and hypertension (Ye et al. 2006; Sachse and Wolf 2007; Billet et al. 2008; Crowley et al. 2008; Koitka and Tikellis 2008; Schmid et al. 2008; Stegbauer and Coffman 2011). Angiotensin II is well known to increase oxidative stress mainly through the activation of nicotinamide adenine dinucleotide phosphate (NADPH) oxidases, one of the key enzymes involved in the generation of reactive oxygen species (ROS) (Garrido and Griendling 2009; Harrison and Gongora 2009). An inappropriate increase in ROS can further potentiate renal vasoconstriction, vascular damage, tubular injury, and fibrosis. Moreover, there is strong data suggesting that ROS accumulation causes further tissue damage by inactivating nitric oxide (NO), production of other cytotoxic and vasocontrictive compounds (i.e., peroxynitrite), and worsening hypertension (Harrison and Gongora 2009; Montezano and Touyz 2012). Since ACE2 is abundantly expressed in the kidney, we sought to examine whether ACE2 is involved in the regulation of oxidative stress in the kidney during baseline conditions.

## Materials and Methods

### Experimental animals

The generation of ACE2 knockout mice (ACE2KO) has been previously described (Gurley et al. 2006). Inbred mice were backcrossed for 10 generations on a C57BL/6 background. Experiments were performed in male ACE2KO mice 20–35 weeks of age along with their age‐matched, wild‐type (WT) littermates. All animals were bred, housed, and maintained in an Association for Assessment and Accreditation of Laboratory Animal Care International‐accredited animal facilities at the Durham Veterans Affairs, Duke University, and the Center for Comparative Medicine at Northwestern University, according to National Institute of Health Guidelines for Care and Use of Laboratory Animals. Approval for animal care and experiments was granted by the Institutional Animal Care and Use Committees of each institution.

### Assessment of oxidative stress

#### NADPH oxidase activity

NADPH oxidase activity in kidney cortex was measured using lucigenin (5 *μ*mol/L) and NADPH (100 *μ*mol/L) as previously described (Block et al. 2007). Light emission was recorded every 1 min over a 10‐min period at room temperature in a 20/20^n^ luminometer (Turner Biosystems, Sunnyvale, CA). Superoxide anion production was expressed as relative chemiluminescence (light) units (RLU)/*μ*g protein. There was no measurable activity in the absence of NADPH. Protein content was measured using the BCA protein assay reagent (Pierce, Rockford, IL).

#### Superoxide measurement

For DHE fluorescence, dihydroethidium (DHE), an oxidative fluorescent dye, was used to measure superoxide (O2^−^) levels in kidney tissues from ACE2KO and WT mice as previously described Nakane et al. (2000). Briefly, 10 *μ*m fresh frozen tissue sections were washed with PBS and then incubated at 37°C for 30 min with DHE (20 *μ*mol/L) in HBSS. In situ fluorescence was captured using a Zeiss confocal laser‐scanning microscope (LSM 510, Dusseldorf, Germany). Four to five random kidney cortex fields were photographed for each mouse and the intensity of the fluorescence was measured using Image J (National Institutes of Health, Bethesda, MD) software. Day‐to‐day variability in DHE staining intensity was controlled by performing studies in a paired fashion whereby an ACE2KO and a corresponding WT kidney were scanned and evaluated each day in parallel.

#### Hydrogen peroxide (H_2_O_2_) production

The production of H_2_O_2_ in fresh tissue from both ACE2KO and WT mice was measured using 100 *μ*mol/L Amplex^®^ Red H_2_O_2_ Assay Kit (Molecular Probes, Eugene, OR). Kidneys were harvested and approximately 2–3 mg of each tissue was placed in separate wells of a 96‐well plate. The plate was incubated at room temperature in working solution containing Amplex Red reagent (10‐acetyl‐3,7‐dihydroxyphenoxazine), 0.2 U/mL horseradish peroxide (HRP) and Krebs‐Henseleit Hepes buffer (118 mmol/L NaCl, 24.9 mmol/L, NaHCO_3_, 11.1 mmol/L Glucose, 5 mmol/L HEPES, 4.6 mmol/L KCL, 1 mmol/L KH_2_PO_4_, 1 mmol/L CaCl_2_, 1.1 mmol/L MgSO_4_, pH 7.4). Kidney tissues were incubated, protected from light, for 15 min. Fluorescence was measured using a microplate reader according to the protocol provided by the manufacturer. Background fluorescence was subtracted from each value, and each measurement normalized to the protein content in each sample (BioRad, Berkeley, CA). H_2_O_2_ release was expressed as micromoles per minute per milligram of protein.

#### Urinary 8‐isoprostane

We collected 24‐h urine samples from control and ACE2 KO mice in individual metabolic cages (Hatteras Instruments, Cary, NC). Urine samples were centrifuged briefly to remove particulate matter and then immediately alliquoted (with the antioxidant butylated hydroxytoluene in ethanol) and frozen at −80°C until assay. 8‐isoprostane levels were measured using a specific competitive immunoassay (Cayman Chemical, Ann Arbor, MI).

#### Kidney catalase and superoxide dismutase (SOD) activity

Catalase and SOD activities in kidney cortex homogenates were measured using commercial kits from Cayman Chemical (Ann Arbor, MI) and Cell Biolabs (San Diego, CA), respectively, as per manufacturers' instructions. The procedure for preparing tissue homogenates was to assay total SOD activity (mitochondrial and cytosolic). Briefly, a portion of kidney cortex was homogenized in a buffer containing: 20 mmol/L KH2PO4, pH 7.0, 1 mmol/L EGTA, 1 mmol/L phenylmethylsulfonyl fluoride, 10 μg/mL aprotinin, and 0.5 μg/mL leupeptin. Homogenates were centrifuged at 800 × ***g*** at 4°C for 10 min to remove the unbroken cells and debris. Protein content in the supernatant was measured using the BCA protein assay reagent (Pierce).

### Enzymatic ACE2 activity

Kidney ACE2 activity was determined following incubation with the intramolecularly quenched synthetic ACE2‐specific substrate Mca‐APK‐Dnp (Anaspec, Fremont, CA). The measurements were performed in black 96‐well plates with a 100 μL total volume. Briefly, 1 μg total protein from renal cortex tissue homogenate was added to wells containing a buffer (50 mmol/L 4‐morpholineethanesulfonic acid, 300 mmol/L NaCl, 10 μmol/L ZnCl2, and 0.01% Triton‐X‐100, pH 6.5), containing EDTA‐free tablets (Roche, Indianapolis, IN) and 10 μmol/l substrate. Reactions were in duplicates (one of two wells constituted a blank). Blank wells contained the same components, but 10 μmol/L of a specific ACE2 inhibitor, MLN‐4760 (gift from Millennium Pharmaceuticals, Cambridge, MA; currently GL1001, Ore Pharmaceuticals, Cambridge, MA) was also added. Fluorescence was followed at ambient temperature for 1 h using the FLX800 microplate fluorescence reader (BIOTEK Instruments Inc., Winooski, VT) at 320 nm excitation and 420 nm emission wavelength. Total fluorescence after subtracting blank values was corrected for protein content.

### Kidney angiotensin II degradation

Kidney cortex lysates (from ACE2 KO and WT mice) corresponding to 6 μg of total protein were individually incubated with 10^−9^mol/L angiotensin II (Sigma‐Aldrich, St. Louis, MO) by constant shaking for up to 24 h at 37°C in PBS pH 7.4 containing 5 μmol/L ZnCl_2_. The incubation was performed with or without addition of the specific ACE2 inhibitor, MLN‐4760 (10^−6^mol/L). Following the incubation, samples were diluted 1/5 in EDTA‐containing buffer (0.1 mol/L phosphate buffer, pH 7.4 containing 0.15 mol/L NaCl, 1 mmol/L EDTA, 0.1% BSA) and immediately frozen. The quantity of Ang II in the diluted samples was measured using enzyme‐linked immunosorbent assay kits (SPIBio, Cayman Chemical). Angiotensin quantity in each sample was expressed as a percentage ratio of the Ang remaining after a given incubation time to the initial Ang load (100%).

### Measurement of endogenous angiotensin II and angiotensin (1–7)

The measurement of angiotensin II and angiotensin (1–7) was performed as previously described Ye et al. (2012). Both plasma and whole kidney angiotensin peptides were extracted using reverse phase phenyl silica columns (50 mg; Thermo Scientific, Pittsburgh, PA) as per manufacturer's instructions. Quantification of Ang II and Ang (1–7) in the extracts was determined using a commercial EIA kit (Cayman Chemical, Ann Arbor, MI and Bachem, San Carlos, CA), as per manufacturer's instructions. Results were reported in fmol/mL (plasma) and in fmol/mg protein (kidney).

### Blood pressure measurements in conscious mice by radiotelemetry

Blood pressures were monitored in conscious, unrestrained mice using a radiotelemetry system as described previously Butz and Davisson (2001), Gurley et al. (2006). Briefly, mice were anesthetized with isoflurane and a pressure‐sensing catheter was implanted into the aorta via the left carotid artery (Butz and Davisson 2001). The transducer unit was then inserted into a subcutaneous pouch along the right flank. Before measurements were recorded, mice were given 1 week to recover from surgery and regain normal circadian rhythms. During the measurement period, mice were housed unrestrained in individual cages, in a quiet monitoring room in the animal facility. Blood pressures were measured continuously over a 10‐sec interval every 5 min and recordings were collected, stored, and analyzed using Dataquest ART software (version 4.1; Transoma Medical, St. Paul, MN).

### Pharmacological blockade of angiotensin receptors

ACE2KO and control mice at the age of 20–30 weeks were treated with losartan (Triangle Compounding Pharmacy, Cary, NC) or placebo for 2 weeks. Losartan was administered to ACE2KO at two doses: 10 mg/kg/day (*n *=* *4) and 30 mg/kg/day (*n *=* *5) in drinking water in two separate experiments. Another group of ACE2KO (*n *=* *6) and WT controls (*n *=* *5 and *n *=* *10) were given vehicle drug, with similar taste and consistency. Water bottles were weighed daily to document accurate drug consumption. At the end of 2 weeks of treatment with losartan, we proceeded to measure markers of oxidative stress as described above.

A separate group of male ACE2 knockout mice at 35 weeks of age underwent either sham operation (*n *=* *8) or osmotic minipump (Alzet 1002) implantation to infuse the AT2 receptor blocker, PD123319 (2 mg/kg/day, *n *=* *8) for 14 days. Pumps were implanted subcutaneously on the back between the shoulder blades and hips while animals were anesthetized by inhalation of isoflurane anesthetic. At the end of 2 weeks of treatment with PD123319, we proceeded to measure markers of oxidative stress as described above.

### Statistical analysis

The values for each parameter within a group are expressed as the mean ± standard error of the mean (SEM). For comparisons between WT and ACE2‐deficient groups, statistical significance was assessed using an unpaired 2‐tailed Student's *t* test. A paired 2‐tailed Student's *t* test was used for comparisons within groups. *P*‐values less than 0.05 were considered significant.

## Results

### ACE2 deficiency is associated with increased markers of oxidative stress in the kidney

**Figure 1:**
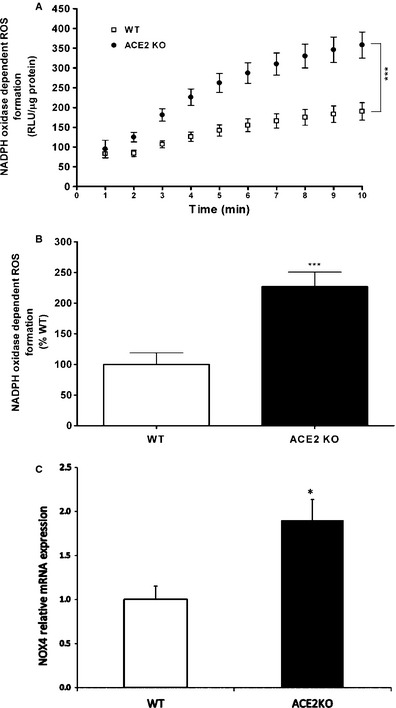
Increased NADPH oxidase activity and superoxide production in ACE2‐deficient mice. (A) Kidney NADPH oxidase activity was significantly increased in ACE2 KO mice as determined by repeated measures general linear model analysis. (B) NADPH oxidase activity expressed as normalized percent of WT control (***P <* *0.001). (C) Nox4 subunit mRNA expression measured by RT‐qPCR in a different group of mice was twofold increased in ACE2KO mice as compared to WT controls (*P <* *0.01).

The main mechanism by which Ang II has been shown to induce oxidative stress is by activation of NADPH oxidases, (Rajagopalan et al. 1996) one of the most important generators of superoxide (˙$O_{2}^{-}$). Since ACE2KO mice have impaired metabolism of Ang II, we measured NADPH oxidase activity in kidney cortex during baseline conditions. NADPH oxidase activity is expressed both as relative chemiluminescent light units (*RLU*)/μg of protein/min and as normalized percent of WT controls (Fig. 1A and B, respectively). In ACE2KO mice, kidney cortex NADPH oxidase activity is significantly higher than WT controls (227 ± 24%, *n* = 6, vs. 100 ± 19%, *n* = 10, respectively, *P* < 0.001). As shown in Fig. 1C, the mRNA expression of *Nox4* in kidney cortex of ACE2KO mice was increased nearly twofold (1.89 ± 0.2 vs. 1 relative units, *P* < 0.05) compared to WT controls, which suggests that the overactivity seen in NADPH oxidase is related to increased *Nox4* expression. Activation of NADPH oxidases can lead to increased production of superoxide. Thus, we measured superoxide levels in the kidneys of ACE2KO and WT mice using DHE staining to assess this effect. Quantitative evaluation of four to five kidney cortex fields per each mouse reveals a significant increase in superoxide levels in ACE2‐deficient mice as compared to WT controls (35.9 ± 4.5 vs. 31.3 ± 3.6 relative densitometry units, respectively, *P* < 0.05; data not shown). Taken together, these data support increased NADPH‐mediated oxidative stress in kidney tissue of mice deficient in ACE2.

**Figure 2:**
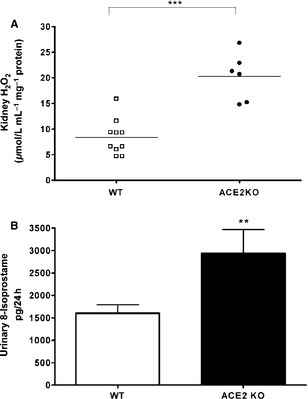
Increased markers of oxidative stress. (A) Kidney cortex H2O2 release in ACE2 KO mice (*n *=* *6) was significantly higher as compared to WT control (*n *=* *10) (20.2 ± 4 vs. 8.41 ± 3 *μ*mol/L/mg/min ***P <* *0.01). (B) Twenty‐four‐hour Urinary 8‐Isoprostane excretion was higher in ACE2 KO mice compared to WT littermates (2939.8 ± 532 vs. 1603.3 ± 186 pg/24 h; **P <* *0.05, *n *=* *5 ACE2 KO,* n *=* *7 WT).

The enzyme superoxide dismutase catalyzes the dismutation of superoxide into hydrogen peroxide (H_2_O_2_) and oxygen (O_2_), and measurement of H_2_O_2_ is one assay that estimates superoxide levels. As shown in Fig. 2A, during baseline conditions kidney H_2_O_2_ production is significantly increased in ACE2KO mice as compared to WT controls (20.2 ± 4 vs. 8.41 ± 3 *μ*mol/L/mg/min, *P *< 0.01, respectively), consistent with the elevated superoxide levels observed.

A 24‐h urine collection was obtained from ACE2 KO and WT mice to assess total excretion of urinary 8‐isoprostane, a compound produced by free radical‐mediated peroxidation of lipoproteins (Montuschi et al. 2004). Loss of ACE2 is also associated with increased level of total urinary 8‐Isoprostane excretion (2939.8 ± 532 vs. 1603.3 ± 186 pg/24 h; *P* < 0.05, *n *=* *5 ACE2KO, *n *=* *7 WT) (Fig. 2B).

These data clearly show that there is significant accumulation of both superoxide and hydrogen peroxide in kidney tissue from ACE2KO mice compared to WT littermates. The elevated level of reactive oxygen species in the kidneys of these mice is likely due to increased ROS production, as reflected by an increased kidney NADPH oxidase activity.

### Loss of ACE2 does not affect catalase and superoxide dismutase activity

Oxidative stress can be caused by an increased production of reactive oxygen species, decreased scavenging mechanisms, or both. Since oxidative stress in ACE2KO mice was increased, we next investigated if an impaired scavenging system could also contribute to the accumulation of reactive oxygen species in mice lacking ACE2. For these experiments, we measured both superoxide dismutase (SOD) and catalase activities in kidney cortex.

**Figure 3:**
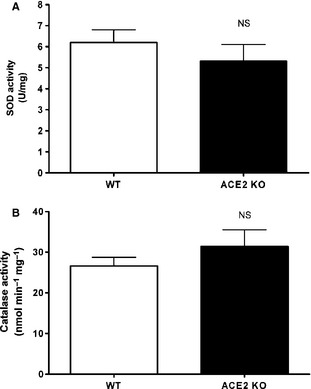
(A) Superoxide Dismutase (SOD) activity in kidney was not different between ACE2 KO (*n *=* *6) and WT (*n *=* *10) mice (5.3 ± 0.8 vs. 6.2 ± 0.6 U/mg protein, *P* = NS). (B) ACE2‐deficient mice (*n *=* *6) exhibited kidney catalase activity levels similar to those of WT mice (*n *=* *10) (31.4 ± 4.1 vs. 26.6 ± 2.1 nmol/min/mg protein, *P* = NS, respectively).

Activity of the antioxidant enzyme superoxide dismutase in kidney is not significantly different between ACE2KO and WT mice (5.3 ± 0.8 vs. 6.2 ± 0.6 U/mg protein, *P* = NS, respectively) (Fig. 3A). ACE2‐deficient mice also exhibit a similar kidney catalase activity as WT mice (31.4 ± 4.1 vs. 26.6 ± 2.1 nmol/min/mg protein, *P* = NS, respectively) (Fig. 3B). These findings suggest that ROS accumulation in kidney during baseline conditions is mainly due to increased production, as a result of NADPH oxidase activation, and not due to alterations in the scavenging system.

### Effects of ACE2 deficiency on Ang II degradation

**Figure 4:**
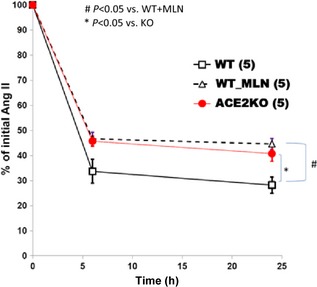
Ex vivo Ang II degradation by kidney cortex preparation from ACE2 KO (*n *=* *5) and WT mice (*n *=* *5). After incubating kidney cortex lysates with exogenous Ang II (10^−9^mol/L) there was an increased degradation of Ang II in WT (*n *=* *5) as compared to ACE2 KO (*n *=* *5) at 6 and 24 h of incubation. Co‐incubation of WT lysates (*n *=* *5) with ACE2 inhibitor, MLN‐4760, ameliorated these differences.

ACE2 hydrolyzes Ang II with high catalytic efficiency and is regarded as a brake on RAS activity (Vickers et al. 2002; Gurley et al. 2006; Wysocki et al. 2010). We hypothesized that the increased markers of oxidative stress seen in our ACE2KO mice is likely due to impaired metabolism of Ang II, ensuing accumulation of this peptide and subsequent NADPH oxidase‐mediated generation of ROS. To test this, lysates of kidney cortex from WT and ACE2KO mice were incubated ex vivo with exogenous Ang II (10^−9^mol/L) to examine the relative contribution of ACE2 enzymatic activity to the hydrolysis of this peptide within the kidney. ACE2 activity levels measured in kidney preparations from each group using a fluorogenic Mca‐APK‐Dnp substrate showed a complete lack of ACE2 enzymatic activity in ACE2KO as compared to WT mice (means: 0.2 ± 1 vs. 108 ± 13 RFU/μg protein/h, *P* < 0.001, respectively) as expected. Furthermore, kidney lysates from WT mice degrade significantly more Ang II than the lysates obtained from ACE2KO (*P* < 0.05) (Fig. 4). Co‐incubation of the WT lysates with a selective ACE2 inhibitor, MLN‐4760, is also associated with a significant decrease in Ang II degradation rate (*P* < 0.05 vs. WT without MLN‐4760) and similar to that of the ACE2KO mice, demonstrating that the differences in Ang II degradation are attributable to ACE2 (Fig. 4). These data show that ACE2 deficiency is associated with less efficient degradation of exogenous Ang II than in WT controls within the kidney cortex ex vivo. Despite differences in rates of Ang II metabolism between ACE2 KO and WT mice, however, there was no significant difference in the levels of plasma and renal Ang II at baseline (see below).

### Effects of ACE2 deficiency on blood pressure and plasma and kidney angiotensin peptides

**Figure 5:**
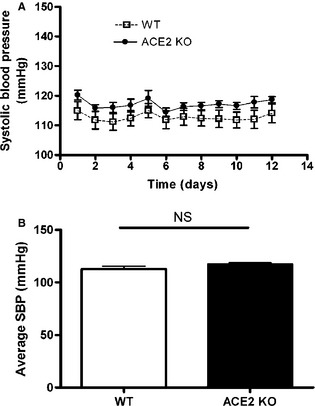
Twenty‐four‐hour mean systolic blood pressure. Systolic blood pressures (SBPs) were recorded continuously in WT (open squares) and ACE2 KO (filled circles) mice. Baseline SBPs were similar in both group (ACE2 KO: 117.13 ± 3 versus WT 112.69 ± 1 mmHg; *P* = 0.186, *n *=* *6).

Using radiotelemetry, we compared the blood pressures between inbred C57BL/6 ACE2KO mice and their WT controls. Baseline blood pressures were measured for approximately 2 weeks in conscious, unrestrained mice. As shown in Fig. 5, both groups had similar systolic blood pressures at baseline. ACE2KO mice had a small trend toward higher systolic blood pressures compared to their wild‐type littermates; however, it did not reach statistical significance (24‐h mean systolic blood pressures: ACE2 KO 117.13 ± 3 vs. WT 112.69 ± 1 mmHg; *P* = 0.186). This is in accordance with prior experiments, where we found a modest elevation of systolic blood pressures in a group of C57BL/6 ACE2‐deficient mice as measured by tail cuff manometry (128 ± 3 mmHg vs. 121 ± 1 mmHg; *P *<* *0.05) (Gurley et al. 2006). Thus, the impact of ACE2 deficiency on baseline blood pressures in inbred C57BL/6‐ACE2 KO mice are modest.

**Figure 6:**
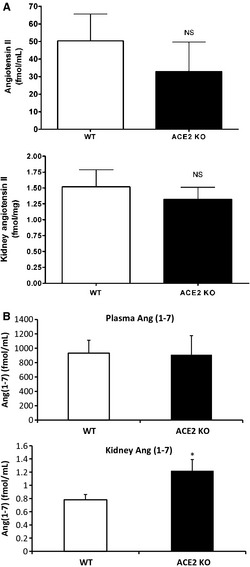
Plasma and Kidney angiotensin II and angiotensin (1–7) levels. (A) No difference between plasma angiotensin II in ACE2 KO and WT mice. Whole kidney samples of ACE2 KO showing levels of Ang II also not different from those of the WT mice. (B) No difference between plasma angiotensin (1–7) in ACE2KO and WT mice. Whole kidney samples in ACE2KO showing levels of Ang(1–7) significantly higher than in the WT mice (*P < 0.05; *n* = 10 WT, *n* = 6 ACE2KO).

Similarly, ACE2‐deficient mice had baseline plasma Ang II levels that were not significantly different from WT mice (32.9 ± 16.8 vs.50.3 ± 15.3 fmol/mL, respectively, *P* = NS). Kidney Ang II levels in ACE2KO mice also did not differ significantly from those in WT mice at baseline (1.32 ± 0.27 vs. 1.52 ± 0.19 fmol/mg protein; *P* = NS) (Fig. 6A). Ang(1–7) levels in plasma were almost identical between ACE2KO and WT mice at baseline (910 ± 265 vs. 932 ± 178 fmol/mL, *P* = NS, respectively). And in the kidney, Ang(1–7) levels were actuallysignificantly higher in ACE2KO as compared to WT mice (1.22 ± 0.17 vs. 0.78 ± 0.08 fmol/mg, *P* < 0.05, respectively) (Fig. 6B). Our data suggest that during baseline conditions, ACE2 deficiency leads to elevated oxidative stress in the kidney, and this seems to occur in the absence of overt hypertension and without major alterations on Ang II or Ang(1–7) levels.

### Effect of angiotensin receptor blockade on kidney NADPH oxidase activity in ACE2‐deficient mice

Our data suggest that, during baseline conditions, ACE2KO mice have increased NADPH oxidase activity associated with increased markers of oxidative stress despite normal blood pressures and angiotensin peptide levels. However, ACE2KO mice did exhibit decreased degradation of Ang II ex vivo as compared to WT controls, which suggests a dynamic effect of ACE2 loss on Ang II availability. In order to test the hypothesis that the increased oxidative stress seen in ACE2KO mice is mediated by Ang II, we reasoned that the administration of either AT1 or AT2 receptor blockers would normalize the NADPH oxidase overactivity seen in kidney cortex of ACE2KO, to a similar level of their WT littermates.

**Figure 7:**
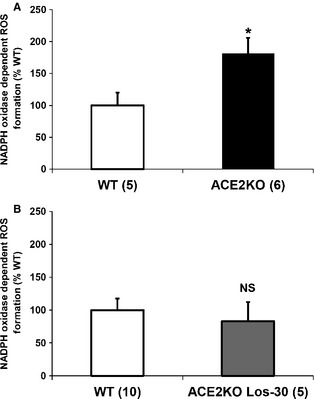
Effect of AT1R blocker losartan on NADPH oxidase activity in ACE2‐deficient mice. Kidney NADPH oxidase activity expressed as percent of WT controls. (A) ACE2 KO mice (black bar; *n *=* *6) after 2 weeks of vehicle have significantly higher levels of NADPH oxidase activity than vehicle‐treated WT mice (*n *=* *5; white bar). (B) ACE2KO mice after 2 weeks of losartan administration at 30 mg/kg/day (*n *=* *5, gray bar) show no elevation of kidney NADPH oxidase activity as compared to vehicle‐treated WT mice (*n *=* *10, white filling); *P <* *0.05 versus WT; NS, not significant.

In keeping with the experiments described above (see Fig. 1), ACE2 KO mice receiving vehicle for 2 weeks also had significantly higher kidney NADPH oxidase activity than vehicle‐treated WT mice (181 ± 24% vs.100 ± 20%, respectively, *P* < 0.05; Fig. 7A). The administration of losartan (10 mg/kg/day; *n *=* *4) for 2 weeks did not decrease kidney cortex NADPH oxidase activity in ACE2KO mice, which remained significantly elevated as compared to vehicle WT mice (201 ± 22% vs.100 ± 20%, respectively, *P* < 0.05). Higher dose of losartan (30 mg/kg/day; *n *=* *5) for 2 weeks, however, normalized kidney cortex NADPH oxidase activity levels in ACE2KO mice compared to WT mice (83 ± 29% vs.100 ± 18%, respectively, *P* = 0.709; Fig. 7B).

In a separate set of ACE2KO mice of similar age, the compound PD123319 (2 mg/kg/day), a specific inhibitor of the AT_2_ receptor, was infused subcutaneously for 2 weeks to examine whether kidney NADPH oxidase activity up‐regulation in ACE2‐deficient mice is related to Ang II overactivity via an AT2R‐dependent mechanism. Kidney NADPH oxidase activity in ACE2‐deficient mice receiving AT2R blocker, PD123319, was not significantly different from that of age‐matched, sham‐operated ACE2KO mice (83 ± 17% vs.100 ± 21%, respectively, *P* = 0.541). Our pharmacological studies suggest that the NADPH oxidase hyperactivity seen in ACE2KO mice is due to Ang II‐mediated activation of the AT1 receptor.

## Discussion

ACE2 is a monocarboxypeptidase that metabolizes Ang II, the major effector peptide of the renin–angiotensin system (RAS) (Donoghue et al. 2000; Tipnis et al. 2000). ACE2 is highly expressed in kidney and heart, which suggests that it may play an important role in renal and cardiovascular function. By studying mice with targeted disruption of the *Ace2* gene, we have shown that ACE2 deficiency is associated with increased markers of oxidative stress during baseline conditions. We have also shown that ACE2 deficiency does not alter the main ROS scavenging pathways within the kidney cortex. Specifically, catalase and superoxide dismutase activities in the kidney were unaffected by genetic ACE2 ablation. This suggests that the increase in reactive oxygen species (˙$O_{2}^{-}$ and H_2_O_2_) seen in ACE2KO is mainly a result of increased NADPH oxidase activity .

We hypothesized that the effect of ACE2 deficiency on oxidative stress would be primarily attributable to increased levels of Ang II in the circulation and/or the kidney. Although the levels of Ang II both in plasma and in the kidney were not significantly increased in ACE2KO mice, our studies showed that kidney cortex lysates from ACE2KO mice degraded Ang II less efficiently than those from WT mice. Moreover, the administration of an AT1 receptor blocker suppressed the hyperactivity of kidney NADPH oxidase seen in ACE2KO mice dose‐dependently whereas AT2R blockade did not. This suggests that ACE2 affects ROS by a mechanism which is, at least in part, AT1R‐dependent.

It has been previously suggested that ACE2 deficiency leads to downregulation of AT1 receptor expression, likely as a counter to hypertensive effects of ACE2 loss and/or Ang II accumulation (Oudit et al. 2006). In kidney samples from patients with diabetic nephropathy or glomerulonephritis, noted to have high intrarenal Ang II concentrations, Wagner et al. found significantly low AT1 receptor mRNA expression (Wagner et al. 1999). In order to rule out the possibility of significant differences in AT1A receptor expression between ACE2KO and WT mice, we performed real‐time quantitative PCR on kidney cortex of both groups, and found that the *Agtr1a* receptor mRNA expression was not statistically different between the two groups (data not shown). The similar *Agtr1a* gene expression suggests that the differences observed in the oxidative stress phenotype are not due to alterations in levels of the AT1A receptor.

ACE2 not only promotes the metabolism of Ang II but also favors the generation of Ang(1–7) (Wysocki et al. 2010). This heptapeptide, acting through the Mas receptor, exerts vasodilatory, anti‐inflammatory, antifibrotic, and atheroprotective effects (Allred et al. 2000; Chappell et al. 2001). In our experiments, we found that both circulating and kidney levels of Ang II were similar in ACE2KO mice as compared to WT. This may suggest that other degradation pathways like aminopeptidase A, dipeptidyl‐aminopeptidase I–III, neprilysin and other peptidases also play a role on renal Ang II metabolism. Interestingly, there was no difference on plasma Ang(1–7) between groups, whereas kidney levels of this peptide were unexpectedly higher in ACE2KO mice. However, since Ang(1‐7) levels were unexpectedly higher in ACE2KO mice, our findings cannot be attributed to reduced formation of Ang(1‐7). These findings suggest that the effect of ACE2 on ROS generation, similar to the effect of this enzyme in blood pressure regulation, is largely Ang II mediated and independent of Ang(1–7). It is important to acknowledge that angiotensin‐converting enxyme (ACE) and other endopeptidases such as neprilysin (Allred et al. 2000; Chappell et al. 2001), can effectively metabolize Ang(1–7) to form Ang(1–5) and Ang(1–4), respectively. This may explain why decreased Ang(1–7) levels were not detected in ACE2 deficicent mice. It should also be noted that the administration of human recombinant ACE2 has been shown to ameliorate oxidative stress in diabetes (Song et al. 2013), consistent with an ACE2‐dependent effect. Regardless of the underlying mechanism whereby ACE2 protects against oxidative stress, our ACE2KO mouse line provides a model to study kidney oxidative stress in disease states such as diabetes or CKD without the need to infuse exogenous Ang II or concerns regarding overt cardiac dysfunction.

We reasoned that examining Ang II degradation ex vivo would provide more functional information than endogenous levels of Ang II alone. Since ACE2 in the kidney is heavily expressed in proximal tubules (Ye et al. 2006; Wysocki et al. 2013). we measured ACE2 activity in kidney cortex from ACE2 KO and WT mice. Our results show that ACE2 deficiency is associated with significantly decreased Ang II degradation in renal tissue ex vivo, and that the accumulation of this peptide is ACE2‐specific, since the effects are reproduced when kidney cortex from WT animals are treated ex vivo with an ACE2 inhibitor, MLN‐4760. A substantial proportion (~50%) of Ang II, however, is degraded in kidney cortex lysates despite the lack of ACE2, whether genetically disrupted (ACE2 KO) or pharmacologically inhibited (WT + MLN‐4760). As stated above, this shows that degradation of Ang II still occurs through other ACE2‐independent metabolic pathways as recently shown by our group Wysocki et al. (2013).

There is abundant evidence that Ang II increases NADPH oxidase activity (Gorin et al. 2004; Harrison and Gongora 2009; Massey et al. 2012) and accordingly, the augmented activity of this enyme in the kidney of ACE2KO mice could result from increased activity of Ang II, due to its delayed degradation. To address whether basal NADPH oxidase activity involves Ang II activation of its receptors, we performed studies using AT1 receptor blocker (losartan) and AT2 receptor blocker (PD123319) in ACE2KO mice. These studies show that the increased kidney NADPH oxidase activity in ACE2KO mice is related to stimulation of AT1 receptors as a result of alterations in metabolism of Ang II.

Oxidative stress causing tissue damage can result from either an increased production of ROS or deficient scavenging mechanisms. Although ROS play important physiological roles (cell growth and proliferation, endothelial regulation, (Miura et al. 2003) inflammatory signals, and immune system), these metabolites are also implicated in the pathogenesis of several conditions, including hypertension (Nakazono et al. 1991; Schnackenberg et al. 1998; Agarwal et al. 2004; Alvarez et al. 2008; Montezano and Touyz 2012). Zhong et al. (2011) previously reported that after 4 days of Ang II infusion in ACE2KO C57BL/6 mice there is an increase in NADPH oxidase activity, as compared to WT, but baseline data in the absence of Ang II infusion were not reported. Elevated Ang II levels in the kidney after infusion of high levels of Ang II were reported in this previous study and also by our group during Ang II hypertension Wysocki et al. (2010). In mice lacking ACE2, an increase in Ang II after such an exogenous infusion is expected owing to the critical role of this enzyme in the degradation of this peptide and the high delivered Ang II dose (Gurley et al. 2006; Wysocki et al. 2010; Ye et al. 2012). The goal of this study was to determine the prevailing effects of impaired degradation of Ang II under baseline conditions and whether this impacts NADPH oxidase‐mediated ROS. Athough in our experiments, which were conducted at baseline, neither plasma nor kidney Ang II levels were elevated in mice with complete ACE2 deficiency, AT1 receptor blockade normalized kidney cortex NADPH oxidase activity dose‐dependently.

Increased ROS production has been involved in multiple pathological processes, including hypertension (Harrison and Gongora 2009). There is strong evidence suggesting that elevated blood pressure can promote ROS accumulation in the kidney and thereby further exacerbate hypertension (Harrison and Gongora 2009). We had previously reported, using tail cuff manometry, that C57BL/6 mice have a modest baseline systolic blood pressure difference of ~7 mmHg as compared to WT littermates (Gurley et al. 2006). However, the 7‐mmHg difference in arterial BP between inbred C57BL/6 ACE2KO and WT mice did not reach statistical significance using radiotelemetry (Gurley et al. 2006). Therefore, in C57BL/6‐ACE2‐deficient mice, a minimal or very modest elevation in baseline blood pressure may exist. Yet a marked increase in kidney ROS is readily apparent and thus cannot be directly attributed to overt hypertension.

In summary, genetic ablation of ACE2 in mice is associated with increased kidney ROS due to elevated levels of NADPH oxidase activity even with only a minimal elevation in baseline blood pressure. While the levels of Ang II are not elevated in the face of complete ACE2 deficiency, the capacity for Ang II degradation is reduced, and the NADPH overactivity is abrogated with the administration of an AT1 receptor blocker, suggesting that the increased ROS seen in this model is AngII‐dependent. Although our ACE2KO model does not allow for the determination of local effects of ACE2 deficiency, we believe that the increased oxidative stress during baseline conditions seen in these mice constitute a unique phenotype due to global deficiency, which reflects the integrated effects of this enzyme on renal physiology. Moreover, the ACE2KO should provide a model to study the effect of ROS overactivity on kidney function and CKD disease progression without the administration of Ang II and in the absence of overt hypertension.

## Conflict of Interest

None declared.
